# Long-term knowledge and skills retention following Helping Mothers Survive and Helping Babies Survive training in Tanzania: a mixed-methods follow-up study

**DOI:** 10.3389/fpubh.2026.1824835

**Published:** 2026-06-22

**Authors:** Beatrice Erastus Mwilike, Ruchius Philbert, Martha Rimoy, Lucy Mabada, Nicodem Komba, Tom Okade, Feddy Mwanga, Rashid Gosse, Joel Ambikile

**Affiliations:** 1 Department of Midwifery, Child and Reproductive Health, Muhimbili University of Health and Allied Sciences, Dar es Salaam, Tanzania; 2 Department of Pediatrics and Child Health, Muhimbili National Hospital, Dar es Salaam, Tanzania; 3 Tanzania Midwives Association (TAMA) Headquarters, Dar es Salaam, Tanzania; 4 Department of Disease Control and Environmental Health, Makerere University School of Public Health, Kampala, Uganda; 5 Elevate Research and Health Services Limited, Kampala, Uganda; 6 Department of Clinical Nursing, Muhimbili University of Health and Allied Sciences, Dar es Salaam, Tanzania

**Keywords:** Helping Babies Survive, Helping Mothers Survive, knowledge retention, maternal and neonatal health, post-training evaluation, Tanzania

## Abstract

**Background:**

The Tanzanian Midwives Association (TAMA), in collaboration with development partners, implemented the *50,000 Happy Birthdays* project (2018–2020) to improve healthcare providers' knowledge and skills in saving lives at birth through the *Helping Mothers Survive* (HMS) and *Helping Babies Survive* (HBS) training modules. Despite large-scale implementation of HMS and HBS training in Tanzania, limited evidence exists on long-term retention of knowledge and skills across multiple modules and on contextual determinants influencing sustainability.

**Methods:**

A mixed-methods cross-sectional design was used to assess knowledge and skills retention by comparing post-test and follow-up test scores among providers in selected health facilities in the Tanga, Geita, and Katavi regions. Four knowledge areas were evaluated using written tests, and five skill areas were assessed using observational checklists. Quantitative data were analyzed using IBM SPSS version 25, employing descriptive statistics, *t*-tests, and one-way ANOVA, with significance set at *p* < 0.05. Thematic analysis was applied to qualitative data to identify factors influencing knowledge and skills retention.

**Results:**

A total of 210 respondents participated; over half held diplomas (*n* = 116; 55.2%) and worked in urban areas (*n* = 123; 58.6%). The largest knowledge decline was observed in pre-eclampsia management (−21.0 points), followed by essential care for small babies (−14.7), controlling bleeding after birth (−12.5), while helping babies breathe showed the smallest decline (−7.7). Skills also showed significant reductions across all domains, with the greatest decline in administration of magnesium sulfate (−17.1) and active man-agement of the third stage of labor (−17.0). Rural providers showed greater declines in key obstetric skills: manual removal of the placenta and active management of the third stage of labor (*p* < 0.05). One-way ANOVA showed that professional education level was significantly associated with knowledge retention only for pre-eclampsia management [F_(2, 205)_ = 6.919, *p* = 0.001, Ω^2^ = 0.049]. A significant difference in skills decline was also observed for nasogastric tube feeding across education levels [F_(2, 205)_ = 3.601, *p* = 0.029, Ω^2^ = 0.020].

**Conclusions:**

Knowledge and skills retention declined significantly over time. Regular refresher training is recommended, particularly for rural healthcare providers and those with lower educational qualifications.

## Introduction

Despite sustained national and global efforts to improve maternal and neonatal health services, maternal and neonatal mortality remain major public health concerns, particularly in low- and middle-income countries (LMICs). Every 2 min a woman dies from preventable pregnancy-related causes, about 800 deaths daily, 95% of which occur in LMICs (1), where limited knowledge and skills among birth attendants are key contributing factors (2). Newborn mortality mirrors this challenge: approximately 6,500 newborns die each day within the first 28 days of life, with 2.3 million deaths reported in 2022 alone (3), largely due to suboptimal quality of care around birth and inadequate skilled care in the immediate postnatal period (4).

Although HMS and HBS programs have been widely implemented, the sustainability of knowledge and skills retention particularly across multiple modules and diverse facility settings remains insufficiently understood in Tanzania. The maternal mortality ratio in Tanzania has sharply declined from 556 deaths per 100,000 live births in 2015/2016 to 104 in 2022 (5), showing progress toward SDG 3.1. However, neonatal mortality has remained nearly stagnant over the past two decades, decreasing only marginally from 25 to 24 deaths per 1,000 live births between 2015/2016 and 2022 (5), highlighting slow progress toward SDG 3.2.2 (1). Although investments in health infrastructure have expanded access to reproductive, maternal, newborn, and child health (RMNCH) services (5), a persistent shortage of skilled health personnel continues to undermine care quality. Indeed, Tanzania faces an estimated 50% shortage of skilled health workers (6), including SBAs despite evidence that up to two-thirds of maternal deaths and almost half of newborn deaths could be prevented by competent, well-trained SBAs (7).

In-service training has been widely used to strengthen provider knowledge and skills in maternal and newborn care (8). Among these, the Helping Mothers Survive (HMS) and Helping Babies Survive (HBS) programs have gained prominence as cost-effective, context-appropriate approaches addressing leading causes of maternal and neonatal mortality (9). Numerous studies have shown that these trainings can improve provider knowledge, skills, and confidence (10), although evidence on clinical practice outcomes remains mixed, with some studies demonstrating improvements and others reporting no significant changes (11). While short-term gains following HMS/HBS training are well documented (12), evidence consistently shows that these gains decline over time, with substantial drops in knowledge and skills observed within 9–10 months post-training (5). This indicates that training alone is insufficient and underscores the need for structured follow-up strategies (5). Although previous studies have identified factors such as training frequency, clinical exposure, cadre, and training modality as potential influences on retention (13), existing findings are fragmented, context-specific, and do not sufficiently explain why long-term retention remains poor in Tanzania despite widespread rollout of HMS/HBS programs. Importantly, there is limited empirical evidence assessing retention across multiple HMS/HBS modules simultaneously, or identifying facility- and provider-level determinants of retention within large-scale national training initiatives.

To address these gaps, this study examined long-term knowledge and skills retention among healthcare providers who received HMS and HBS training under the 50,000 Happy Birthdays (50KHB) program implemented in Tanzania from 2018 to 2020. This post-training follow-up survey evaluates retention across four knowledge areas and five skills domains and identified factors influencing long-term outcomes. By providing a comprehensive assessment of retention patterns within a large national cohort, this study contributes new evidence to inform design of sustainable training and follow-up strategies to improve maternal and newborn care. Furthermore, this study advances literature in three key ways: (1) by simultaneously assessing retention across multiple HMS and HBS modules; (2) by integrating quantitative decline patterns with qualitative system-level explanations; and (3) by examining retention within a large-scale national training initiative rather than small pilot implementations.

## Materials and methods

### Study setting

This study was conducted in six purposively selected districts across three mainland regions of Tanzania—Geita, Tanga, and Katavi—where the *50,000 Happy Birthdays* (50KHB) project had previously been implemented and evaluated. The selected districts were Geita Town Council, Chato District Council, Tanga City Council, Muheza District Council, Mpanda District Council, and Mpanda Town Council. In each district, three health facilities of varying levels (hospital, health center, and dispensary) were randomly selected from among those that had participated in the 50KHB program. Facility selection in each region was guided by the following criteria: (i) Facilities with providers who had already received training on *Helping Mothers Survive* (HMS) and *Helping Babies Survive* (HBS) modules; (ii) Inclusion of a mix of facility types (hospital, health center, and dispensary); and (iii) Representation of both urban and rural contexts within each region.

### Study design

This study employed a mixed-methods cross-sectional design to assess the long-term retention of knowledge and skills among healthcare providers who had previously received Helping Mothers Survive (HMS) and Helping Babies Survive (HBS) training under the 50,000 Happy Birthdays (50KHB) program. The quantitative component assessed current knowledge and skills retention by comparing follow-up assessment scores with the original post-training endline scores from the 50KHB program. Standardized HMS and HBS evaluation tools were used, including multiple-choice knowledge assessments and Objective Structured Clinical Evaluations (OSCEs). Data collection was supported by trained master trainers in each region to ensure consistency and adherence to standardized procedures. In addition to the quantitative assessment, a qualitative descriptive approach was used to explore contextual factors influencing knowledge and skills retention among healthcare providers. Qualitative data were collected through facility-level interviews, focus group discussions, clinical observations, and desk reviews to obtain in-depth insights into perceived barriers and facilitators affecting retention in clinical practice. The quantitative component of the study was reported in accordance with the Strengthening the Reporting of Observational Studies in Epidemiology (STROBE) guidelines, while the qualitative component followed the Consolidated Criteria for Reporting Qualitative Research (COREQ) checklist to ensure transparency in data collection, analysis, and reporting. Completed STROBE and COREQ checklists are provided as Supplementary materials. Ethical approval for this study was obtained from the Muhimbili University of Health and Allied Sciences Research and Ethics Committee (Ref. No. DA.282/298/01.C/). Participation was voluntary, and all participants provided written in-formed consent.

### Study participants

This study involved participants across both quantitative and qualitative components.

#### Quantitative component

Healthcare providers from selected health facilities who had been trained in the HMS and HBS modules.

#### Qualitative component

Focus groups with selected Healthcare providers, In-depth interviews with training implementation support team including district/regional level coordinators [District and regional reproductive coordinators (DRCHCOs), maternity ward in-charges and child health coordinators RRCHCOs)] and Tutors from selected nursing and midwifery training institutions.

### Inclusion and exclusion criteria

#### Inclusion criteria

Participants were eligible if they:

- Were currently residing in the Geita, Tanga, or Katavi regions;
- Belonged to one of the three defined target populations;
- Had previously received HMS and HBS training through the 50,000 Happy birthdays project.
- Provided informed consent to participate in skills drills, focus group discussions, or interviews.

#### Exclusion criteria

Participants were excluded if they were on study leave or sick at the time of data collection.

### Sampling methods and procedure

#### Quantitative sampling

**Figure 1:**
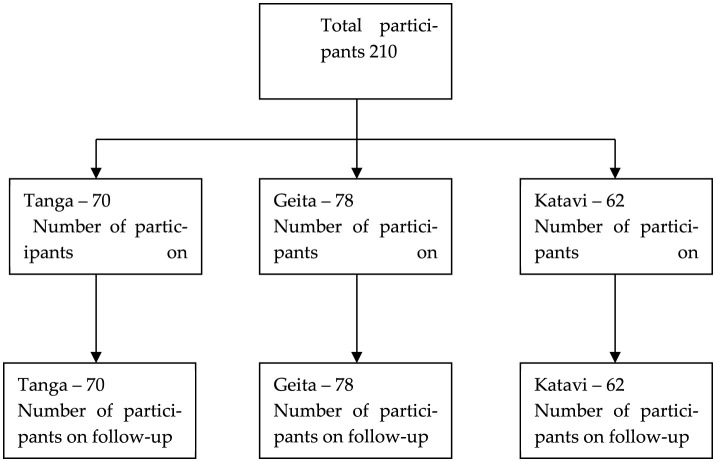
Flow diagram shows number of participants between post-testing and the follow-up assessment.

All healthcare providers and tutors who had received HMS and HBS training and were working in the 28 purposively selected health facilities or three training institutions were approached and invited to participate in the study. Those who consented were consecutively recruited until all eligible participants had been enrolled. A total of 210 participants were successfully recruited from all selected study sites (Table 1). The study sample was limited to facilities that had implemented the training and to participants who had fully completed it. In each region, we selected two districts, one rural and one urban, as shown in Table 1. Participant continuity between post-test and follow-up assessments was verified using training attendance records, facility staffing registers, and confirmation from facility leadership. Unique identifiers, including participant names, cadre, facility, and district, were used to match individuals across the two time points. These records were cross-checked with facility in-charges during data collection to confirm participant identity. Only participants whose records could be reliably matched to the original post-test data were included in the paired analysis (Figure 1).

**Table 1:** Sample of the study sites.

Region	Tanga	Geita	Katavi
**Urban district**	**Tanga town council**	**Geita town council**	**Mpanda municipal council**
Education institution	Tanga COHAS	Geita school of nursing	n/a
Hospital	Bombo Regional Referral Hospital	Geita regional refferal hospital	Katavi Referral Regional Hospital
Primary health facilities	Ngamiani HC, Mikanjuni HC, Duga dispensary	Nyankumbu HC, Kasamwa HC, Bung'wankoko dispensary	Town council clinic, Kakese dispensary, Mwamkulu dispensary
**Rural district**	**Muheza district council**	**Chato district council**	**Mpanda district council**
Education institution	St.Augustine Muheza IHAS	n/a	n/a
Hospital	Muheza DH	Chato DH	n/a
Primary health facilities	Mkuzi HC, Bulwa HC, Misozwe dispensary, Mkanyageni dispensary	Bwanga HC, Buzilamoyo dispensary, Butalama dispensary, Kibehe dispensary	Mwese HC, Mishamo HC, Kalema HC, Katuma dispensary, Sibwesa dispensary, Kasekeseke dispensary

### Sampling for qualitative part

Qualitatively, focus group discussions (FGDs) and in-depth interviews (IDIs) were conducted with purposively selected participants to assess the factors affecting knowledge and skills retention. FDs included a purposively selected subset of trained healthcare providers from the quantitative sample. Similarly, tutors and district/regional coordinators [District Reproductive and Child Health Coordinators (DRCHCOs), maternity ward in-charges, Regional Reproductive and Child Health Coordinators (RRCHCOs)] were purposively selected for the IDIs because of their roles in supporting the implementation, delivery, and supervision of the HMS/HBS training program. As key stakeholders involved in training and mentorship, they provided valuable system-level perspectives on factors affecting knowledge and skills retention. Providers were selected from diverse backgrounds to gather feedback reflecting varied socio-demographic profiles. Final sample sizes were determined by theoretical saturation, the point at which no new concepts emerged from the data collected from a diverse sample in terms of relevant characteristics and experiences. Theoretical saturation was reached after conducting three focus groups, each consisting of 12 participants. Additionally, a total of 16 in-depth interviews were conducted across the three regions.

### Data collection methods and procedures

Data collection occurred from January to March 2023, following the online post-test survey on HMS/HBS knowledge and skills that was conducted in May 2020 for the 50,000 Happy Birthdays project. The assessment of healthcare providers' knowledge and skills employed a mixed-methods approach, combining qualitative and quantitative techniques, including in-depth interviews, Objective Structured Clinical Examinations (OSCE), and questionnaires. Qualitative data from interviews and focus group discussions were audio-recorded, transcribed verbatim, and translated from Kiswahili into English. An interview guide was used to ensure consistency during qualitative data collection. Data were collected by a team of principal researchers. Interviews lasted approximately 30 to 60 min and were conducted in Kiswahili. Quantitative data were collected using tools originally developed for the pre- and post-tests of the *50,000 Happy Birthdays* program intervention. Paper questionnaires were administered to participants after obtaining signed informed consent. Data collection was performed by researchers and trained research assistants. The research team conducted the interviews were not members of the study community. The assessment tools were designed to evaluate knowledge and skills retention related to HMS and HBS among healthcare providers and tutors. The evaluation focused on two HMS modules, *Bleeding After Birth Complete* (BABC) and *Pre-Eclampsia/Eclampsia* (PEE), and three HBS modules, *Helping Babies Breathe* (HBB 2.0), *Essential Care for Every Baby* (ECEB), and *Essential Care for Small Babies* (ECSB). The knowledge assessment tools included socio-demographic questionnaires and multiple-choice questions covering the training topics. The skills assessment tools included socio-demographic information, instructions for conducting skills demonstrations, and observation checklists.

Specifically, the knowledge tests for HMS modules included:

(a) Helping Mothers Survive: Bleeding After Birth Complete Knowledge Assessment
(b) Helping Mothers Survive: Pre-Eclampsia and Eclampsia Knowledge Test

The skills tests for HMS included OSCE assessments on:

(a) Active Management of the Third Stage of Labor (AMTSL)
(b) Shock Management
(c) Administering the loading dose of Magnesium Sulfate (MgSO4)

The knowledge tests for HBS modules included:

(a) Essential Care for Every Baby Knowledge Check
(b) Essential Care for Small Babies Knowledge Check

The skills tests for HBS modules included assessments on:

(a) Helping Babies Breathe
(b) Care for Small Babies

### Integration

We used an explanatory sequential mixed-methods design, where the quantitative findings guided the qualitative exploration of contextual factors. The integration of both strands occurred during interpretation, using triangulation and narrative weaving to connect the results. In the Discussion, we further highlight this integration by linking observed declines in knowledge and skills to qualitative explanations, such as gaps in equipment, lack of mentorship, and staff rotation.

### Data management and analysis

#### Quantitative data analysis

Quantitative data from questionnaires were first entered into an Excel database and then imported into IBM SPSS Statistics version 25 for analysis. Descriptive statistics—including frequencies and measures of central tendency—were used to assess levels of knowledge and skills retention at follow-up. Because the study did not include a control group, the analysis focused exclusively on within-provider changes over time, rather than estimating the causal impact of the training itself or comparing retention against expected baseline decay in knowledge. All demographic data are summarized in Table 2. Model assumptions were evaluated using standardized residuals from the one-way ANOVA. Residual diagnostics indicated that residuals were approximately symmetrically distributed with means close to zero and no evidence of extreme skewness or kurtosis. Although Shapiro-Wilk tests on standardized residuals were statistically significant for some outcomes, this was expected given the large sample size (*n* = 210) and the sensitivity of formal normality tests. Visual inspection of histograms and Q-Q plots did not suggest substantial departures from normality.

**Table 2:** Demographic characteristics of participants (*n* = 210).

Characteristics	Frequencies *n* (%)
Sex	
Male	65 (31%)
Female	145 (69%)
Age	
Mean age	35.7
SD	7.5
Educational level	
Certificate (EN)	76 (36.2%)
Diploma (RN/AMO)	116 (55.2%)
Degree (RN/MD)	16 (7.6%)
Others (MA/CA)	2 (1%)
Health facility	
Dispensary	37 (17.6%)
Health center	63 (30%)
Hospital	110 (52.4%)
Region	
Tanga	70 (33.3%)
Geita	78 (37.1%)
Katavi	62 (29.5%)
Districts/council	
Tanga city	46 (21.9%)
Muheza DC	24 (11.4%)
Geita MC	47 (22.4%)
Chato DC	31 (14.8%)
Mpanda MC	
Tanganyika DC	

Group sizes were explicitly examined and reported. For analysis by residence, 123 participants worked in health facilities located in urban areas and 87 in rural areas. For analysis by level of professional education, participants were categorized as certificate holders (*n* = 76), diploma holders (*n* = 116), and degree holders (*n* = 16); the ‘others' category (*n* = 2) was omitted from the ANOVA due to insufficient cell size. Homogeneity of variances was assessed using Levene's test, and Welch's ANOVA was applied. For comparisons involving two groups (e.g., urban vs rural), one-way ANOVA was consistently applied and reported using F-statistics to ensure uniformity in statistical reporting across analyses. Given the focus on comparisons of group means and the robustness of ANOVA to moderate departures from normality under these conditions, one-way ANOVA was considered appropriate. Effect sizes were reported to aid interpretation of the findings. In this study, the primary objective was to assess overall knowledge and skill retention over time. Accordingly, the primary analyses focused on the overall knowledge and overall skill scores, which we had pre-specified as the primary outcomes. Analyses of individual knowledge and skill domains were treated as secondary, exploratory analyses intended to provide additional context on specific areas of performance. Because these secondary outcomes were exploratory and aimed at informing future refinements to the training rather than supporting confirmatory inference, the secondary analyses were considered hypothesis-generating. Consequently, *p*-values were interpreted cautiously, and no formal adjustments for multiple testing were applied in this study. This study primarily focused on within-provider comparisons over time, which reduces confounding related to individual baseline characteristics, but does not eliminate potential time-varying confounding factors such as differences in clinical exposure, mentorship, workload, and facility context. Although some of these factors were not quantitatively measured, they were explored through the qualitative component of the study. Selected variables, including residence (urban/rural) and level of professional education, were examined using stratified analyses (one-way ANOVA), and no multivariable adjustment was performed.

### Qualitative data analysis

A deductive–inductive thematic analysis approach was employed to identify, analyze, and interpret patterns within the qualitative data. Audio recordings were transcribed verbatim, and transcripts in Kiswahili were prepared. Researchers repeatedly read the translated transcripts while cross-referencing with the original audio recordings to ensure accuracy. Meaningful units of information were identified and assigned codes. These codes were then grouped into themes, which were further categorized into sub-themes. The data were subsequently translated and presented in textual form. To ensure validity, triangulation, member checking, and collaboration with other investigators were applied during the qualitative analysis. Discrepancies among coders were resolved through an iterative consensus process. After independently coding the transcripts, coders compared their coding decisions and discussed any differences during scheduled team meetings. When disagreements arose, the team revisited the relevant excerpts together until consensus was reached. In cases where alignment could not be achieved, a senior researcher reviewed the excerpts and provided a final decision. This process helped ensure consistency, rigor, and credibility in the qualitative analysis.

## Results

### Characteristics of the study participants

A total of 210 healthcare providers participated in the study. The mean (SD) age was 35.7 (7.5) years, and most participants were female (*n* = 145; 69.0%). The majority held a diploma (*n* = 116; 55.2%), followed by certificate holders (*n* = 76; 36.2%). Just over half (n = 110; 52.4%) were employed at regional or district referral hospitals. The region with the highest number of participants was Geita (*n* = 78; 37.1%), followed by Tanga (*n* = 70; 33.3%). Geita and Tanga municipal councils contributed the largest shares of participants, with 47 (22.4%) and 46 (21.9%), respectively. Regarding residential area (data not shown in the table), more than half of the participants (*n* = 123; 58.6%) worked in health facilities located in urban areas (Table 2).

### Participants' knowledge retention scores

**Figure 2:**
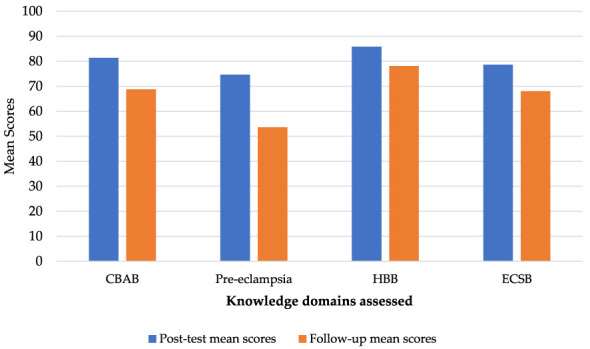
Mean scores across different knowledge domains.

Four knowledge areas were assessed, including two related to maternal care controlling bleeding after childbirth and managing pre-eclampsia and two related to newborn care helping babies breathe and essential care for small babies. Participants' mean follow-up knowledge scores (SD) were highest for helping babies breathe [78.1 (12.4)] and lowest for managing pre-eclampsia [53.6 (14.4)] (Figure 2).

Follow-up knowledge scores were compared to post-test knowledge scores (previously assessed), and the change in mean (SD) scores was computed to determine knowledge retention. The results showed a decline in mean knowledge scores across all areas, with the greatest decrease observed in managing pre-eclampsia [−21.0 (15.0)] and the smallest in helping babies breathe [−7.7 (13.0)]. A paired t-test was performed to assess the differences between follow-up and post-test mean knowledge scores to determine whether the observed declines were statistically significant. For all knowledge areas assessed, there was a significant decrease in knowledge scores. Specifically, statistically significant drops were observed in mean knowledge scores for controlling bleeding after birth (*t* = 15.404, *p* < 0.001), pre-eclampsia (*t* = 20.276, *p* < 0.001), helping babies breathe (*t* = 8.580, *p* < 0.001), and essential care for small babies (*t* = 19.620, *p* < 0.001) (Table 3).

**Table 3:** Paired dependent *t*-test analysis of post-test and follow-up knowledge scores (Means, standard deviations, and significance values).

Area assessed	Post-test		Follow-up		Difference		Paired ***t***-test		
	Mean	SD	Mean	SD	Mean	SD	95%CI	*t*	*p* value
CBAB	81.3	7.3	68.8	10.5	−12.5	11.7	−14.05678, −10.86703	15.404	<0.001
Pre-eclampsia	74.6	8.1	53.6	14.4	−21.0	15.0	−23.01567, −18.93671	20.276	<0.001
HBB	85.8	7.8	78.1	12.4	−7.7	13.0	−9.45166, −5.91977	8.580	<0.001
ECSB	78.6	7.3	64.0	11.5	−14.7	10.8	−16.12989, −13.18439	19.620	<0.001

CBAB, Controlling bleeding after birth; HBB, Helping Babies Breathe; ECSB, Essential Care for Small Babies; MRP, Manual removal of placenta; AMTSL, Active management of the third stage of labor; AdMgSO4, Administration of magnesium sulfate; FNGT, Feeding with a naso-gastric tube; CI, Confidence interval; HMS, Helping Mothers Survive; η^2^, Eta squared.

A one-way ANOVA was performed to compare the mean knowledge drop scores between participants working in urban and rural areas. Participants working in rural areas had a statistically significantly higher drop in mean knowledge scores for managing pre-eclampsia [F_(1, 208)_ = 4.491, *p* = 0.035] and for helping babies breathe [F_(1, 208)_ = 5.005, *p* = 0.026] (Table 4).

**Table 4:** One-way ANOVA results showing mean differences in knowledge scores by residence (including F-statistic and significance values).

Measure	Urban		Rural		F (1, 208)	Sig.	η2	Cohen's *d*	95% CI for *d*
Knowledge domain	Mean	SD	Mean	SD					
CBAB	−13.3	11.7	−11.2	11.7	1.677	0.197	0.008	−0.18	−0.45, 0.10
Pre-eclampsia	−22.8	15.7	−18.4	13.7	4.491	0.035	0.021	−0.30	−0.57, −0.02
HBB	−6.0	13.2	−10.0	12.4	5.005	0.026	0.023	0.31	0.03, 0.59
ECSB	−13.7	10.4	−16.0	11.3	2.421	0.121	0.012	0.21	−0.06, 0.49

CBAB, Controlling bleeding after birth; HBB, Helping Babies Breathe; ECSB, Essential Care for Small Babies.

Another statistical analysis performed to assess knowledge decline was a one-way ANOVA to determine differences in mean knowledge scores among participants according to their levels of professional education (i.e., certificate, diploma, and degree). There was a statistically significant difference in mean knowledge scores for the management of pre-eclampsia across levels of professional education (F = 6.919, *p* = 0.001). *Post hoc* analyses were conducted to examine pairwise differences in mean knowledge scores for the management of pre-eclampsia across levels of professional education [F_(2, 205)_ = 6.919, *p* = 0.001, Ω^2^ = 0.049). The results indicated statistically significant differences between certificate and diploma holders (*p* = 0.003), and between certificate and degree holders (*p* = 0.020). No significant differences were found in knowledge variance between diploma and degree holders. Although statistically significant declines in knowledge were observed, effect sizes ranged from small to moderate, suggesting variability in practical impact (Table 5).

**Table 5:** One-Way ANOVA results showing mean differences in knowledge scores across education levels (Including F-statistic and significance values).

Measure	Knowledge domain	Sum of squares	Mean square	F (2, 205)	*p* value	Ω^2^	95% CI for Ω^2^
CBAB	Between groups	285.954	142.977	1.041	0.355	0.000	0.000, 0.010
	Within groups	28,157.104	137.352				
	Total	28,443.058					
Pre-eclampsia	Between groups	2,954.150	1,477.075	6.919	**0.001**	**0.049**	**0.049, 0.100**
	Within groups	43,763.158	213.479				
	Total	46,717.308					
HBB	Between groups	473.955	236.977	1.412	0.246	0.000	0.000, 0.010
	Within groups	34,407.103	167.840				
	Total	34,881.058					
ECSB	Between groups	304.682	152.341	1.295	0.276	0.001	0.000, 0.010
	Within groups	24,109.583	117.608				
	Total	24,414.264					

CBAB, Controlling bleeding after birth; HBB, Helping Babies Breathe; ECSB, Essential Care for Small Babies. Values in bold indicate statistical significance at the 5% level (*p* < 0.05).

### Participants' skills retention scores

**Figure 3:**
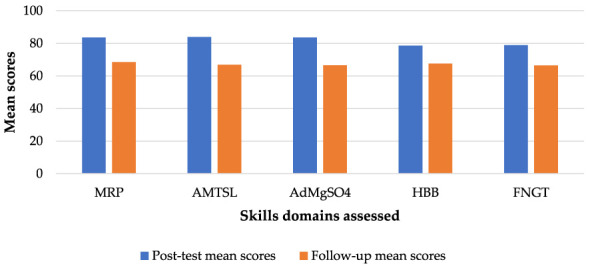
Mean scores across different skill domains.

Five skills areas were assessed, including three related to maternal care—manual removal of the placenta, active management of the third stage of labor, and administration of magnesium sulfate—and two related to newborn care—helping babies breathe and feeding with a nasogastric tube. Participants' follow-up mean skills scores (SD) were highest for manual removal of the placenta [68.5 (16.1)] and lowest for feeding with a nasogastric tube [66.4 (14.6)] (Figure 3). The results showed a decline in mean skills scores in all areas assessed, with the largest drop observed in the administration of magnesium sulfate [−17.1 (18.7)] and the smallest in helping babies breathe [−11.1 (11.0)].

A paired *t*-test was performed to assess differences between follow-up mean skills scores and post-test mean skills scores to determine whether the changes were statistically significant. In all skills areas assessed, there was a statistically significant decline in scores. Specifically, there was a significant drop in mean skill scores for manual removal of the placenta (*t* = 13.427, *p* < 0.001), active management of the third stage of labor (*t* = 15.840, *p* < 0.001), administration of magnesium sulfate (*t* = 13.210, *p* < 0.001), helping babies breathe (*t* = 14.609, *p* < 0.001), and feeding with a nasogastric tube (*t* = 16.113, *p* < 0.001) (Table 6).

**Table 6:** Paired dependent *t*-test analysis of post-test and follow-up skills scores (Means, standard deviations, and significance values).

Skill domain	Post-test		Follow-up		Difference		Paired *t*-test		
	Mean	SD	Mean	SD	Mean	SD	95%CI	*t*	*p* value
MRP	83.6000	7.88688	68.4905	16.07912	−15.1095	16.30723	−17.32793, −12.89112	13.427	<0.001
AMTSL	83.9952	8.15255	66.9571	14.47694	−17.0381	15.58795	−19.15865, −14.91754	15.840	<0.001
AdMgSO_4_	83.6268	9.49162	66.6048	20.56920	−17.0766	18.68859	−19.62506, −14.52805	13.210	<0.001
HBB	78.6190	7.54747	67.5095	14.45178	−11.1095	11.02031	−12.60871, −9.61034	14.609	<0.001
FNGT	78.8571	7.13027	66.4048	14.56886	−12.4524	11.19895	−13.97586, −10.92890	16.113	<0.001

A one-way ANOVA was performed to compare the mean skills drop scores between participants working in urban and rural areas. Participants working in rural areas had a statistically significantly higher drop in mean skills scores for manual removal of placenta [F_(1, 208)_ = 14.917, *p* < 0.001] and for active management of third stage of labor [F_(1, 208)_ = 6.271, *p* = 0.013] (Table 7).

**Table 7:** One-way ANOVA results showing mean differences in skills scores by residence (Including F-statistic and significance values).

Skill domain	Urban		Rural		ANOVA				
Measure	Mean	SD	Mean	SD	F (1, 208)	*p* value	η2	Cohen's *d*	95% CI for *d*
MRP	−11.6	15.3	−20.2	16.5	14.917	**<0.001**	0.067	0.54	0.26, 0.82
AMTSL	−14.8	16.1	−20.2	14.3	6.271	**0.013**	0.029	0.35	0.07, 0.63
AdMgSO_4_	−15.4	17.5	−19.4	20.2	2.356	0.126	0.011	0.21	−0.06, 0.49
HBB	−12.1	11.6	−9.7	10.0	2.367	0.125	0.011	−0.22	−0.49, 0.06
FNGT	−13.1	12.1	−11.5	9.8	1.028	0.312	0.005	−0.14	−0.42, 0.13

A One-Way ANOVA was conducted to assess differences in skills drop scores among participants according to their levels of professional education, i.e., holders of certificate, diploma, degree, and others. A statistically significant difference was found only in the skills related to feeding with a nasogastric tube [F_(2, 205)_ = 3.601, *p* = 0.029, Ω^2^ = 0.020]. However, the *post hoc* analysis did not reveal statistically significant pairwise differences between groups, likely due to small group sizes and limited differences between group means rather than adjustment for multiple comparisons (Table 8).

**Table 8:** One-way ANOVA results showing mean differences in skills scores across education levels (including F-statistic and significance values).

Measure		Sum of squares	Mean square	F (2, 205)	*p* value	Ω^2^	95% CI for Ω^2^
MRP	Between groups	1,168.408	584.204	2.218	0.111	0.007	0.000, 0.021
	Within groups	54,006.650	263.447				
	Total	55,175.058					
AMTSL	Between groups	1,295.748	647.874	2.703	0.069	0.011	0.000, 0.025
	Within groups	49,133.862	239.677				
	Total	50,429.611					
AdMgSO4	Between groups	1,204.191	602.095	1.720	0.182	0.002	0.000, 0.015
	Within groups	71,393.636	349.969				
	Total	72,597.826					
HBB	Between groups	224.457	112.228	0.933	0.395	0.000	0.000, 0.010
	Within groups	24,665.524	120.320				
	Total	24,889.981					
FNGT	Between groups	884.224	442.112	3.601	**0.029**	**0.020**	**0.002, 0.045**
	Within groups	25,165.656	122.759				
	Total	26,049.880					

Values in bold indicate statistical significance at the 5% level (*p* < 0.05).

### Factors influencing knowledge and skills retention

A summary of the themes, sub-themes, and representative quotations identified from the qualitative analysis is presented in Table 9.

**Table 9:** Summary table of themes, sub-themes and quotes.

Main theme	Sub-theme	Quotes
Learning materials	Availability of learning materials	“*For example, when you are given a handout and revise it every time, it will help you build experience. Although you may not be able to attend to a patient physically, it will assist you to read the references and help keep your knowledge intact.”* (FGD, Tanga)
	Topic revision in CMEs	“*We normally have learning sessions at our facility; we have a teaching plan. Every Wednesday, we prepare a lesson. Maybe this week it will be eclampsia, next week it will be PPH, and we learn through those sessions, gaining experience.”* (FGD, Tanga)
	Establishment of a learning corner	“*Sometimes, when there is no client and the labor ward is calm, you go to a small room with a learning corner. There we have manikins such as ‘Mama Natalie,' ‘Mama Besiye,' and ‘Mama Yu,' among others. We are able to learn from these manikins. At the end of the day, when you come across a real case or patient, you can recall what you have learned and transfer the knowledge and skills to the real patient.”* (IDI, GRRH)
Mentorship and supportive supervision		“*Mentorship should be done, and supervision from our experts from CHMT is important. When they conduct supervision multiple times, they bring you back to class to review the required processes related to the training.”* (IDI, Kasamwa)
Practice	Frequent practice of the procedure	“*The big thing is that we should not get tired of practicing at all! When you practice a lot, it builds confidence and ability because you have physically done the procedure; it remains in your mind. Therefore, the key is to practice every day.”* (FGD, Muheza)
	Attending cases related to trained topics	“*…it helps retain knowledge when we encounter a case several times. For example, after training on manual removal of the placenta, I attended such a case within the same week and was able to manage it successfully. Repeated exposure helps us keep learning.”* (FGD, Tanga)
Staff rotation/shifting		“*…when shifted to another unit, one loses skills because without practice, you gradually forget.”* (IDI, Katavi) “*Midwives should ideally work in the same maternity unit after training, for example, in helping babies breathe. If they are shifted to surgical or medical wards where those skills are not practiced, retention suffers.”* (IDI, Ilembo) “*To retain skills, one must work in the same unit where training was conducted for at least 6 months. Shifting to OPD or other units leads to loss of skills due to lack of practice.”* (IDI, Kasamwa)
Training		“*…doing sessions every week and on-the-job training help. For example, when we manage an emergency PPH case, we work with fellow providers and teach each other.”* (IDI, Buga) “*I think training should be conducted repeatedly. If one group receives training, then another group should be trained later. On-the-job training builds morale and competence.”* (FGD, Tanga) “*…teaching others helps knowledge stick in your mind, thereby improving competence and the quality of services provided.”* (FGD, Muheza)
Availability of equipment		“*…if you have enough equipment, it helps you provide good services with the required skills. Scarcity of equipment makes it challenging to maintain quality and skills, but sufficient equipment enables proper service delivery.”* (IDI, Buga)

#### Learning materials

***a. Availability of Learning Materials*** Participants reported that the availability of learning materials such as handouts and reference books is a crucial factor for knowledge and skills retention. Learning materials allow learners to review, reinforce, and apply the concepts and skills acquired in the classroom. Handouts and reference books also serve as sources of feedback, guidance, and self-assessment. By having access to learning materials, learners can enhance their understanding, deepen their memory, and transfer their knowledge to new situations.

“*For example, when you are given a handout and revise it every time, it will help you build experience. Although you may not be able to attend to a patient physically, it will assist you to read the references and help keep your knowledge intact.”* (FGD, Tanga).

***b. Topic Revision in CMEs*** Participants highlighted the importance of topic revision during Clinical Medical Education (CME) sessions as a factor enhancing knowledge and skills retention. Topic revision helps learners consolidate their learning and recall it more easily. Moreover, revisiting topics after a gap can strengthen memory traces and make them more resistant to forgetting.

“*We normally have learning sessions at our facility; we have a teaching plan. Every Wednesday, we prepare a lesson. Maybe this week it will be eclampsia, next week it will be PPH, and we learn through those sessions, gaining experience.”* (FGD, Tanga).

***c. Establishment of a Learning Corner*** Participants reported the establishment of a learning corner as another factor for knowledge retention because it provides a space where learners can actively engage with content and review materials at their own pace. A learning corner can also help learners apply their knowledge to real-world scenarios, increasing confidence and competence. Participants described the presence of a learning corner used to refresh their knowledge and skills in maternal and newborn care.

“*Sometimes, when there is no client and the labor ward is calm, you go to a small room with a learning corner. There we have manikins such as ‘Mama Natalie,' ‘Mama Besiye,' and ‘Mama Yu,' among others. We are able to learn from these manikins. At the end of the day, when you come across a real case or patient, you can recall what you have learned and transfer the knowledge and skills to the real patient.”* (IDI, GRRH).

#### Mentorship and supportive supervision

Participants emphasized the need for mentorship and supervision to ensure mastery of content taught during training. Mentorship and supportive supervision help learners learn from experienced mentors, receive constructive feedback, and improve their skills and performance. These processes also foster a culture of learning and collaboration, where providers can share knowledge and insights.

“*Mentorship should be done, and supervision from our experts from CHMT is important. When they conduct supervision multiple times, they bring you back to class to review the required processes related to the training.”* (IDI, Kasamwa).

#### Practice

***a. Frequent Practice of the Procedure*** Participants noted that frequent practice enhances knowledge and skills retention. Regular practice consolidates memory, builds confidence, refines performance, and helps overcome challenges. Without frequent practice, learners may forget procedural steps, lose confidence, and make errors.

“*The big thing is that we should not get tired of practicing at all! When you practice a lot, it builds confidence and ability because you have physically done the procedure; it remains in your mind. Therefore, the key is to practice every day.”* (FGD, Muheza)

***b. Attending Cases Related to Trained Topics*** Attending cases related to the topics they were trained in was reported as a factor influencing knowledge retention and performance. Through case attendance, providers reinforce learning, gain confidence and feedback, and adapt to different contexts.

“*…it helps retain knowledge when we encounter a case several times. For example, after training on manual removal of the placenta, I attended such a case within the same week and was able to manage it successfully. Repeated exposure helps us keep learning.”* (FGD, Tanga).

#### Staff rotation/shifting

Participants indicated that staff rotation or shifting influences knowledge and skills retention both positively and negatively. Rotation assigns healthcare providers to different units or tasks within a facility, which can have benefits but also risks such as loss of hands-on skills, reduced efficiency, and increased training costs due to re-training needs.

“*…when shifted to another unit, one loses skills because without practice, you gradually forget.”* (IDI, Katavi) “*Midwives should ideally work in the same maternity unit after training, for example, in helping babies breathe. If they are shifted to surgical or medical wards where those skills are not practiced, retention suffers.”* (IDI, Ilembo)

“*To retain skills, one must work in the same unit where training was conducted for at least 6 months. Shifting to OPD or other units leads to loss of skills due to lack of practice.”* (IDI, Kasamwa).

#### Training

Training was described as a key factor in knowledge and skills retention, including on-the-job training, teaching others, self-directed revision, and receiving updates on evidence-based practices. Such training increases confidence, competence, and commitment among healthcare providers.

“*…doing sessions every week and on-the-job training help. For example, when we manage an emergency PPH case, we work with fellow providers and teach each other.”* (IDI, Buga)

“*I think training should be conducted repeatedly. If one group receives training, then another group should be trained later. On-the-job training builds morale and competence.”* (FGD, Tanga)

“*…teaching others helps knowledge stick in your mind, thereby improving competence and the quality of services provided.”* (FGD, Muheza).

#### Availability of equipment

Participants highlighted the availability of equipment as influencing knowledge and skills retention because it enables providers to apply learning in practice and improve performance. However, equipment availability alone is insufficient; adequate training must accompany it.

“*…if you have enough equipment, it helps you provide good services with the required skills. Scarcity of equipment makes it challenging to maintain quality and skills, but sufficient equipment enables proper service delivery.”* (IDI, Buga).

## Discussion

This mixed-methods study aimed to assess factors influencing healthcare providers' knowledge and skills retention following the Helping Mothers Survive and Helping Babies Survive training. Regarding knowledge, a significant decline was observed across the four domains assessed: controlling bleeding after childbirth, managing pre-eclampsia, helping babies breathe, and providing essential care for small babies. Similarly, a significant drop occurred in the five skills areas assessed: manual removal of the placenta, active management of the third stage of labor, administration of magnesium sulfate, helping babies breathe, and feeding with a nasogastric tube. Notably, the decline in specific knowledge and skills domains was more pronounced among healthcare providers with lower professional education levels and those working in rural areas. Qualitative findings help explain these quantitative declines, with participants consistently describing limited opportunities for practice, lack of equipment, infrequent mentorship, and staff rotation as barriers to maintaining competence.

Our findings demonstrate an observed decline in knowledge and skills scores among healthcare providers following the Helping Mothers Survive and Helping Babies Survive training, reflecting reduced retention over time rather than a causal effect of the training itself. Similar results were reported in Ethiopia, where midwives' knowledge and skills in helping babies breathe declined 1 year after training (13). Conversely, a study conducted in Tanzania evaluating the Helping Mothers Survive: Bleeding After Birth training found that while knowledge and simulated basic delivery skills decayed after 9 months, providers' confidence and simulated obstetric emergency skills were largely retained, underscoring differing retention patterns across competency domains (13). Consistent with our findings of retention patterns, a study in Mwanza Region, Tanzania, suggest that healthcare workers who received comprehensive RMNH training supplemented with onsite clinical mentorship exhibited strong overall retention of knowledge and performance across a broad range of maternal and newborn care competencies. Additionally, a simulation-based BEmONC training package in rural Tanzania reported sustained high objective skills scores up to 12 months post-training when ongoing facility-based practice and mentorship were present, indicating that structured, contextualized training approaches can enhance long-term retention of knowledge and skills (7). Similarly, a longitudinal evaluation of a clinical mentorship intervention in primary health facilities in Malawi demonstrated that sustained mentorship and supportive supervision contributed to improved maternal and neonatal care performance, further highlighting the importance of post-training support mechanisms in maintaining competencies over time (17).

Various factors influence maternal and neonatal care knowledge and skills retention, including refresher training (7, 8). Recent simulation-based training in Tanzania improved providers' competencies in critical obstetric care, and broader national training initiatives have reported sustained retention and performance in maternal and newborn care up to at least 18 months when supported by ongoing coaching and supervision (14). Our qualitative findings reinforce this, with participants emphasizing that weekly on-the-job training, regular topic revisions during CME, and opportunities to teach peers improved their ability to recall and apply skills.

Participants working in urban areas experienced a significantly higher drop in mean knowledge for managing pre-eclampsia but a significantly lower drop in mean knowledge for helping babies breathe. Conversely, participants in rural areas experienced a significantly higher drop in mean skills for manual removal of the placenta and active management of the third stage of labor. These differences highlight varied knowledge and skills retention challenges across settings. A previous systematic review noted a scarcity of literature comparing professional education, training, and continuous professional development programmes in maternal and newborn care between rural and urban settings, as most studies fail to address how educational opportunities and competency maintenance differ by context (14). Qualitative and service readiness evidence indicates that rural healthcare providers often face *poorer clinical exposure, limited equipment availability, and fewer formal learning opportunities*, which can contribute to sharper declines in skill-based assessments after training. For example, rural facilities in Tanzania showed *fewer training resources* compared to urban counterparts. Additionally, rural workforce research from sub-Saharan Africa highlights ongoing challenges such as *inadequate supervision, poor professional support, and limited continuing education opportunities*, which further disadvantage rural practitioners relative to those in urban settings. These disparities in case exposure, resource availability, and institutional practices help explain why skill retention may be lower and more variable in rural facilities, whereas some urban settings report *more regular CME sessions and structured supervision that support sustained competencies* (14).

Regarding professional education levels, certificate holders showed a significantly higher drop in mean knowledge for pre-eclampsia compared to diploma and degree holders. However, differences in skills retention were not significant. This suggests that knowledge retention decline is more pronounced among certificate holders. This disparity underscores the need for targeted refresher training and supportive supervision for providers with lower educational qualifications. This aligns with a recent Tanzanian study in Zanzibar that found professional qualification (being a medical doctor or assistant medical doctor) and greater clinical experience were significantly associated with higher proficiency in maternal health competencies such as pre-eclampsia management, underscoring how level of education and hands-on experience influence both knowledge and practical skills relevant for retention after training (15). The probable explanation is that healthcare workers with lower education levels may have weaker foundations in medical knowledge and clinical skills, resulting in faster decline of retained information (14). This interpretation is supported by qualitative accounts in which certificate-level providers reported relying more heavily on handouts and learning corners due to limited theoretical background, and noted challenges in retaining complex material without frequent mentorship.

A statistically significant difference in skills decline for manual removal of placenta (MRP) and active management of the third stage of labor were observed between healthcare providers working in urban and rural settings. Providers in rural areas demonstrated a substantially greater reduction in mean skills scores compared to their urban counterparts, indicating a meaningful difference in skill retention between the two groups. This finding suggests that rural-based providers may face greater challenges in maintaining competency in MRP, potentially due to limited opportunities for practice, lower case exposure, and reduced access to mentorship and supportive supervision compared to urban settings. Moreover, qualitative findings suggest potential differences in clinical exposure between facility levels, which may help explain observed patterns, that aligns with evidence from rural Tanzanian studies showing better retention in settings with structured simulation-based reinforcement (16).

Our qualitative findings highlighted several key facilitators of knowledge and skills retention among healthcare providers, including availability of learning materials, frequent practice, mentorship and supportive supervision, on-the-job training, and staff rotation/shifting. The identification of these factors underscores their importance in designing interventions to improve retention. Importantly, integrating the qualitative and quantitative evidence suggests that declines in scores are not simply a function of time since training but reflect systemic constraints including inconsistent practice opportunities, insufficient supervisory structures, and staffing patterns that interrupt skill application. For example, recent studies have shown that midwives' knowledge and practical skills decline over time when workplace environments lack functional equipment and regular opportunities for practice, while those in settings with better supervision and resources retain competencies longer. Similarly, reviews of clinical supervision practices document widespread systemic barriers, such as inadequate supervisor preparation and limited time for mentoring, which impede ongoing skills reinforcement. Together, these findings highlight that retention challenges stem from broader health system limitations rather than the passage of time alone (13, 15), reflecting the multifaceted and context-dependent nature of factors influencing knowledge and skills retention. Therefore, retention should be conceptualized not merely as an educational outcome but as a systems level performance indicator influenced by workload, supervision structures, equipment availability, and workforce deployment policies.

### Study strengths and limitations

This study provides a comprehensive snapshot of knowledge and skills retention following Helping Mothers Survive and Helping Babies Survive training. Standardized procedures for data collection and analysis, including adherence to COREQ for qualitative data and STROBE for quantitative data, enhanced the rigor and credibility of the findings. The results offer valuable insights to inform future evidence-based interventions targeting healthcare providers' knowledge and skills retention. Furthermore, the mixed-methods design allowed triangulation of quantitative and qualitative data, providing complementary insights. We also included participants from both urban and rural settings and across various health facility levels which improved representativeness and enhances external validity within similar settings, but caution is needed when generalizing to regions with substantially different demographic or health system characteristics. Despite these strengths, the current study has some limitations that should be considered when interpreting the findings. Although OSCEs provide standardized assessment of procedural competence, they may not fully capture performance in real clinical settings. The cross-sectional design limited the ability to infer causality, and the absence of a control group prevents direct comparisons, reducing the robustness of conclusions regarding the effectiveness of the training program. Participants were nested within facilities and districts, and the analyses did not formally adjust for clustering, which may have influenced variance estimation. Additionally, although within-provider comparisons reduce confounding from fixed individual characteristics, time-varying factors such as differences in clinical exposure, mentorship, and workload were not quantitatively controlled for and may have influenced the observed outcomes. Future studies using multilevel modeling are recommended. Given the exploratory nature of secondary domain-specific analyses, no formal correction for multiple comparisons was applied. Therefore, these findings should be interpreted cautiously due to an increased risk of type-I error. In addition, although purposive sampling was used to include a diverse range of healthcare providers, the results reflect experiences from selected facilities and regions and may not be generalizable to other institutions or settings.

### Implications for policy and practice

The findings of this study highlight the need to strengthen strategies that sustain knowledge and skills among healthcare providers trained under the HMS/HBS program. Both the quantitative and qualitative results indicated a significant decline in knowledge and skills over time, particularly among midwives working in rural facilities and those with lower professional education levels. To address this, routine refresher trainings should be institutionalized within the national RMNCH training framework, with emphasis on high-impact clinical modules such as management of eclampsia and obstetric emergencies. In addition, simulation-based and case-based learning approaches should be integrated into training programs to enhance practical competency, especially in rural facilities where exposure to certain obstetric complications may be limited. Establishing structured mentorship programs, including onsite supportive supervision and digital mentorship platforms, could further strengthen long-term knowledge and skill retention.

The study also revealed that limited clinical exposure in rural health facilities contributes to skill attrition among trained providers. Introducing staff exchange programs between rural primary health facilities and higher-level referral hospitals could enhance hands-on learning opportunities and reinforce clinical competencies. Additionally, the frequent reassignment of trained staff away from maternity units was identified as a barrier to maintaining HMS/HBS skills. Health system policies should therefore encourage longer retention of trained personnel within maternity departments to ensure continuous application of acquired competencies.

Finally, the availability of essential equipment emerged as a key determinant of effective application of HMS/HBS knowledge and skills. Participants in rural facilities reported that shortages of basic obstetric and neonatal equipment often limit their ability to practice what they had learned during training. Strengthening the national medical supply chain to ensure the provision of minimum RMNCH equipment packages across all delivery facilities is therefore critical. Ensuring equitable distribution of essential equipment between rural and urban health facilities would support healthcare providers in translating their knowledge and skills into improved maternal and newborn health outcomes.

## Conclusions

This study demonstrated a significant decline in both knowledge and skills among healthcare providers following Helping Mothers Survive (HMS) and Helping Babies Survive (HBS) training, highlighting challenges in long-term retention of competencies. The decline was observed across all assessed domains, with greater reductions in specific clinical areas such as pre-eclampsia management and key obstetric skills. Differences in retention were also evident across contextual factors, with providers in rural settings and those with lower levels of professional education experiencing greater losses in selected knowledge and skills areas.

Findings from the qualitative component suggest that retention is influenced not only by time since training but also by health system factors, including limited opportunities for practice, inadequate mentorship and supportive supervision, staff rotation, and insufficient availability of essential equipment and learning resources. These systemic constraints hinder the continuous application and reinforcement of acquired competencies.

To sustain the gains achieved through HMS and HBS training, there is a need for integrated and context-specific strategies. These should include regular refresher training, structured mentorship and supportive supervision, continuous on-the-job learning opportunities, and improved availability of essential equipment. Strengthening these components within the health system is critical to enhancing long-term retention of knowledge and skills and ultimately improving the quality of maternal and newborn care.

## Data Availability

The raw data supporting the conclusions of this article will be made available by the authors, without undue reservation.
