# Supplementary material for: Long-term knowledge and skills retention following Helping Mothers Survive and Helping Babies Survive training in Tanzania: a mixed-methods follow-up study
Source: Front Public Health. 2026 Jun 22;14:1824835. doi: 10.3389/fpubh.2026.1824835 (PMC13333648; doi:10.3389/fpubh.2026.1824835)
Supplement: Supplementary file 1 [file Table_1.docx]

# Table 10: COREQ Checklist Summary

| **COREQ Domain** | **Item** | **How Addressed in This Study** |
| --- | --- | --- |
| Research Team and Reflexivity | Interviewer credentials | Conducted by trained researchers with a medical background |
|  | Interviewer experience and training | Researchers underwent training and had experience with Maternal and Newborn care |
|  | Relationship with participants | The research teams conducting the interviews were not members of the study community, which may have reduced the influence of insider bias on participants’ responses. |
| Study Design | Theoretical framework | Not explicitly theory-driven; exploratory thematic analysis approach used |
|  | Participant selection | Purposive sampling |
|  | Setting | Participants recruited from the facilities to where training was conducted. |
|  | Sample size | Focus groups were organized to include participants with similar characteristics to facilitate open discussion.  Final sample sizes were determined by theoretical saturation on FGDs.  Final sample sizes were determined by thematic saturation on IDIs. |
|  | Non-participation | None reported; all approached participants agreed |
|  | Interview guide | Semi-structured; developed and refined iteratively |
|  | Repeat interviews | Not conducted |
|  | Audio/visual recording | Yes, all interviews audio-recorded with consent |
|  | Field notes | Taken during and after interviews. |
|  | Duration | 30–60 minutes |
|  | Data saturation | Yes, saturation discussed and achieved. In this study, saturation was defined as the point at which no new themes or sub-themes emerged from the data |
| Analysis and Findings | Data transcription and Translation | Verbatim transcription in Swahili; translated to English  All interviews were conducted in Swahili and subsequently translated into English for analysis. Translations were performed by bilingual researchers fluent in both Swahili and English.  Data analyzed using reflexive thematic analysis. |
|  | Data coding | Line-by-line coding; team-based analysis; initial codebook developed collaboratively |
|  | Software used | Microsoft Excel |
|  | Derivation of themes | Inductively developed from coded data.  The analysis was inductive, allowing themes to emerge from the data, and semantic, focusing on the explicit content of participants’ responses. |
|  | Rigor measures (credibility, etc.) | Credibility, dependability, and confirmability ensured through daily debriefings, peer review of coded data and audit trail. |
|  | Participant checking (member checking) | Not conducted due to logistical constraints |
|  | Quotation presentation | Included in Results (representative quotations with anonymized IDs) |
|  | Consistency between data and findings | Ensured through collaborative theme development and iterative analysis |
|  | Clarity of themes | Themes clearly named and structured collaboratively. |
