# Supplementary material for: Long-term knowledge and skills retention following Helping Mothers Survive and Helping Babies Survive training in Tanzania: a mixed-methods follow-up study
Source: Front Public Health. 2026 Jun 22;14:1824835. doi: 10.3389/fpubh.2026.1824835 (PMC13333648; doi:10.3389/fpubh.2026.1824835)
Supplement: Supplementary file 2 [file Table_2.docx]

**Table 10: STROBE Checklist Summary**

| **STROBE Domain** | **Item** | **How Addressed in This Study** |
| --- | --- | --- |
| Title and Abstract | Title |  |
|  | Abstract | Well structured summarizing background, methods, results, and conclusions |
| Introduction | Background | Clarified scientific background including reasoning to conduct a study. |
|  | Objectives | Both primary and secondary objectives of this work are well stated. |
| Methods | Study design | Mixed design well clarified. |
|  | Setting | Clarified locations where implementations were done. |
|  | Participants | Participants recruited from the facilities to where training was conducted. Eligibility criteria and selection process explained. |
|  | Variables | Well clarified , knowledge and skills domains. |
|  | Data sources | Sources of data is clearly started, data collected from human subject. |
|  | Bias | Well clarified including mitigation strategy. |
|  | Study size | Recruited participants clarified including sample size, both on post-testing and follow-up. |
|  | Quantitative variable | Yes, all interviews audio-recorded with consent |
|  | Statistical methods | Analysis procedures well clarified. |
|  | Participants | Flow diagram used to describe participants recruitment process. |
|  | Descriptive data | Demographic information of participants well summarized. |
|  | Outcome data | Changing knowledge and skills scores overtime well presented. |
|  | Main Results | Estimates and CI well presented. |
|  | Other Analysis | Additional tests performed to strengthen presentation of our data. |
| Discussion | Key results | Key results well presented with reference to study objectives |
|  | Limitations | Well clarified including some mitigation strategies. |
|  | Interpretation and comparison | Provided cautious overall interpretation considering objectives, limitations, multiplicity of analyses, and evidence from other studies. |
|  | Generalisability | Aspect of external validity of study results well elaborated. |
